# Adaptability of Electrospun PVDF Nanofibers in Bone Tissue Engineering

**DOI:** 10.3390/polym17030330

**Published:** 2025-01-25

**Authors:** Tereza Havlíková, Nikola Papež, Zdenka Fohlerová, Pavel Kaspar, Rashid Dallaev, Klára Částková, Ştefan Ţălu

**Affiliations:** 1 Department of Physics, Faculty of Electrical Engineering and Communication, Brno University of Technology, Technická 2848/8, 61600 Brno, Czech Republic; tereza.havlikova1@vut.cz (T.H.); papez@vut.cz (N.P.); kasparp@vut.cz (P.K.); rashid.dallaev@vut.cz (R.D.); 2 Department of Microelectronics, Faculty of Electrical Engineering and Communication, Brno University of Technology, Technická 3058/10, 61600 Brno, Czech Republic; fohlerova@vut.cz; 3 Central European Institute of Technology, Purkyňova 656/123, 61200 Brno, Czech Republic; klara.castkova@ceitec.vutbr.cz; 4 Department of Ceramics and Polymers, Faculty of Mechanical Engineering, Brno University of Technology, Technická 2896/2, 61600 Brno, Czech Republic; 5 Directorate of Research, Development and Innovation Management (DMCDI), Technical University of Cluj-Napoca, Constantin Daicoviciu Street, No. 15, 400020 Cluj-Napoca, Cluj County, Romania

**Keywords:** biocompatibility, bone tissue engineering, bone regeneration, cell–substrate interactions, electrospinning, nanofiber fabrication, osteoblasts, piezoelectric polymer, plasma treatment, polyvinylidene fluoride, scaffold

## Abstract

This study focused on the development of a suitable synthetic polymer scaffold for bone tissue engineering applications within the biomedical field. The investigation centered on electrospun polyvinylidene fluoride (PVDF) nanofibers, examining their intrinsic properties and biocompatibility with the human osteosarcoma cell line Saos-2. The influence of oxygen, argon, or combined plasma treatment on the scaffold’s characteristics was explored. A comprehensive design strategy is outlined for the fabrication of a suitable PVDF scaffold, encompassing the optimization of electrospinning parameters with rotating collector and plasma etching conditions to facilitate a subsequent osteoblast cell culture. The proposed methodology involves the fabrication of the PVDF tissue scaffold, followed by a rigorous series of fundamental analyses encompassing the structural integrity, chemical composition, wettability, crystalline phase content, and cell adhesion properties.

## 1. Introduction

With a rising prevalence of myoskeletal disorders due to aging, obesity, and inactivity, effective bone regeneration therapies are needed. Tissue engineering offers a promising alternative to current treatments. Bone tissue engineering (BTE) aims to develop these alternative therapies. Bones, working with muscles, maintain posture and enable movement, while also storing essential growth factors and minerals like calcium and phosphorus [1].

Many studies have documented the importance of the extracellular matrix (ECM) for tissue formation and regeneration. The ECM provides cells with a 3D scaffold structure that mediates cell-to-cell communication and influences cell adhesion, migration, proliferation, differentiation, and the formation of additional ECMs [2,3,4]. These investigations have shown that a universal material will be challenging, if not almost impossible, to find, given that each tissue exhibits different properties and behavioral dynamics [5].

For tissue therapy, a scaffold mimicking the target tissue’s ECM structure and function is crucial to support cell growth and proliferation. The scaffold design, particularly the material and fabrication, must meet criteria relating to the biocompatibility, controlled biodegradability, bioactivity, microarchitecture, and mechanical properties [5,6].

There are a large number of materials for BTE. Namely, these can be metals, bioceramics, natural polymers, synthetic polymers, or composites. Responsive scaffolds in particular are currently becoming very popular. Responsive scaffolds offer the controlled release of growth factors, while mechanical stimulation enhances bone regeneration within these structures. These scaffolds can be activated by and respond to various external stimuli, such as light, magnetism, and the pH, or internal stimuli, including cytokines, enzymes, and biological signals. They can also deliver drugs in a timely manner in response to a diverse array of circumstances [7]. This paper focuses on a synthetic polymer. The advantage of synthetic materials is the possibility to control their chemical and physical properties in the manufacturing process, to specifically and reproducibly fabricate a suitable structure, and if necessary, to functionally modify it. Important biocompatible, non-biodegradable synthetic polymers include, for example, nylon-6, which, in addition to its chemical resistance, is notable for its flexibility and tensile strength but does not have piezoelectric properties. Thus, piezoelectric materials used as responsive scaffolds are coming to the fore in the BTE industry, capable of generating an electrical charge on their surface in response to even small mechanical stresses that can help activate cells [8,9,10].

As an innovative synthetic polymer which is also widely used as a composite, polyvinylidene fluoride (PVDF) can be considered for this purpose [11]. Its chemical formula is (CH_2_–CF_2_)_n_. It is a thermoplastic material that is widely used in many different biomedical applications. This polymeric material has attracted attention due to its significant piezoelectric properties in particular, which make it a potential candidate for osteoinduction. Many studies have been limited to the use of 2D nanofibrous PVDF-based tissue scaffolds for the cultivation of cells in vitro only, and thus a systematic investigation of the cellular response to in vivo electrospun PVDF is still mostly missing [12].

Due to the presence and varying concentration of ions and other charged components in the ECM and on both sides of the cell membranes, such biological structures can be considered electrically active. Through the involvement of certain behavioral signaling pathways, the exchange of ions, nutrients, and growth factors, tissue can be stimulated to heal and regenerate, or specifically in the case of bone, to ossify and osteogenesise. In addition, the surface of bone tissue is considered to be inherently charged, suggesting that the induction and conduction of electrical signals in the proposed tissue scaffold will play an essential role in simulating the natural environment in bone tissue [13]. Achieving precise control over the crystalline structure and phase content of PVDF is crucial for its application in biomedical devices. This requires the optimization of manufacturing processes and storage conditions to maintain the desired properties [14].

Several well-established technologies have been developed to produce tissue scaffolds for use in BTE. Namely, these fabrication methods include decellularization, phase separation, solvent casting and particulate leaching, gas foaming, freeze-drying, 3D bioprinting, and electrostatic spinning, which was used in this research.

Electrospinning, as a method for producing nanofibers for BTE, has drawn considerable interest due to its ability to create structures similar to the naturally fibrous ECM. Polymer electrospun nanofibers may be a noteworthy candidate for designing an adequate structure that can serve as a functional tissue scaffold for cell implantation when combined with a suitable biocompatible material [9,15].

The nanofibers’ large surface area and the nanofiber mesh’s overall porous structure provide an oxygen throughput and promote cell adhesion, proliferation, migration, and differentiation. The nanofiber surface can be modified to achieve desirable properties such as surface hydrophilicity or bioactivity if necessary. The incorporation of additional bioactive elements, such as enzymes or growth factors, may be considered to functionalize the tissue scaffold [15,16,17].

Nanofibers are typically collected into a non-woven disordered membrane during electrospinning. However, natural bone has markedly anisotropic properties manifested by highly oriented ECM fibers and ordered bone cells. The cellular morphology of the settled cells can be modulated through the alignment of the nanofibers, which further affects intercellular communication and the overall tissue function. This modulation is manifested in aligned fibers during cell culturing by the extension of the cytoskeleton and cell nucleus, which become aligned and elongated in the direction of the fibers during growth [17].

Despite extensive research in the BTE industry, autograft transplantation is still the most common method of treating bone defects [18,19]. Tissue engineering generally relies on the proper function and cooperation of the individual cultured cells, the tissue scaffolds, and the flow of signals and materials between them.

In addition to meeting the basic parameters for tissue scaffold design and construction, many other issues need to be resolved, and the resolution of one issue often leads to the creation of another. The most significant technological challenges include the following.

An alternative to mesenchymal stem cells is, for example, embryonic stem cells, which have two remarkable properties: the ability to proliferate in an undifferentiated but pluripotent state and the ability to differentiate into many specialized cell types. In recent years, a method of transforming adult somatic cells into pluripotent stem cells using so-called genetic reprogramming without the use of embryos has also been investigated. However, these cells do not usually participate in the remodeling of mature adult tissue. They may require significant laboratory manipulation and preparation [6].

Osteoinduction is a biological process that stimulates the formation and growth of bone tissue in the body. It involves the stimulation of the differentiation of stem cells into osteoblastic cell lines, mainly through signaling molecules produced by the microenvironment [20].

It was found that the piezoelectric properties of some materials are also related to osteoinduction, as such materials generate electrical impulses under mechanical stress, which can lead to the desired stimulation of cell growth and regeneration. Thus, it is highly desirable to develop a material that, in addition to meeting basic compatibility parameters, has piezoelectric properties such as a high β-phase with the potential to stimulate tissue growth without an external power supply or the injection of stimulating drugs [13,21,22]. In the scientific studies of M. Kitsara et al. [23], osteoblasts cultured on PVDF nanofibers were analyzed through the evaluation of changes in the intracellular calcium concentration over time. Calcium is involved in intracellular signaling and metabolic pathways and participates in signal transmission between cells as part of the activation of the corresponding ion channels. Piezoelectric stimulation was found to induce a measurable calcium influx, which subsequently activated cells, leading to an increase in their growth and differentiation. The results of the study suggested that cells grown on electrospun PVDF tissue scaffolds attached to the nanofibers, generating adhesion forces that resulted in mechanical stress on the nanofibers. This mechanical stress, through the piezoelectric effect, was responsible for the retrograde electromechanical stimulation of the cells themselves (autostimulation).

To sum up, among the important factors that influence how cells will survive on a tissue scaffold, besides the non-toxicity of the material, are the average size of pores in the scaffold and their distribution and connectivity, which together ensure the infiltration of the entire scaffold volume by cells. The architecture of the scaffold, i.e., the nanofiber diameter and alignment, as well as the surface wettability of the material, is important for proper cell adhesion [17,23]. Consideration of the content of the piezoelectric β-phase [23] is also important for exploring the potential of PVDF for future bone cell stimulation [23]. Hence, these challenges will be addressed in the following sections.

This work explores the fabrication of a PVDF material and methods for the design and construction of a compatible tissue scaffold. Their advantages and disadvantages are compared. Polyvinylidene fluoride nanofibers with piezoelectric properties as a promising material and electrostatic spinning technology are explored. Due to their great functional potential, electrospun nanofibers, the types of structures they can form, and the parameters of their fabrication process are discussed in more depth.

The design of the tissue carrier–cell microstructure was developed, and the chosen fabrication procedures are presented. The tissue carrier was subsequently fabricated according to the design and fundamental research analysis of its structure [24]. An investigation of the chemical composition, wettability, nature of the crystalline phases, and cell adhesion was conducted to evaluate the fibers’ adaptability to bone tissue engineering applications. In summary, recommendations for a suitable nanofibrous structure compatible with BTE are provided.

## 2. Material and Methods

This paper evaluates the suitability of several electrospun nanofiber samples made from a PVDF material to create a bone-compatible tissue scaffold. Therefore, the experimental part only dealt with the fabrication and investigation of these nanostructures. The main goal was to fabricate a non-toxic scaffold structure in which cells can survive, incorporate appropriately into the scaffold, and prosper. The material was chosen according to many other parameters beyond the biocompatibility, e.g., the ability to osteoinduce via the piezoelectric effect; however, the morphological, structural, and interaction properties of the tissue scaffold samples were mainly investigated to assess their fundamental adaptability.

Based on the findings, demands, and recommendations in the previous section, Section 1, PVDF meets the requirements to function as a modern responsive scaffold material. For the reasons of sufficient flexibility and porosity, it was produced in the form of nanofibres. The fibers were compared in two fabricated variants due to their compatibility with cells. In order to increase the cell seeding, plasma treatment was subsequently performed. The material was examined, including its crystalline phases. As a final step, representative human cells were cultivated, placed, and investigated on several different types of treated scaffolds.

### 2.1. Preparation of Tissue Scaffold Solution

A 20% PVDF solution (Sigma Aldrich, St. Louis, MO, USA) with a molecular weight of 275.000 g/mol was used. PVDF was dissolved in dimethyl sulfoxide and acetone (DMSO/Ac) prepared in a 7:3 ratio. The resulting mixture was heated for 24 h on a stirrer at 80 °C and 200 rpm before use.

### 2.2. Tissue Scaffold Fabrication by Using Electrospinning

**Figure 1:**
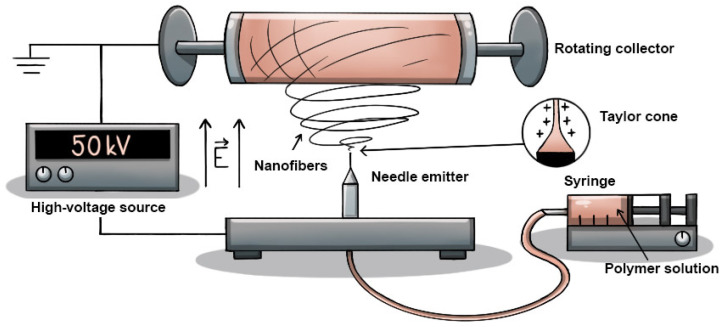
Schematic of the nanofiber fabrication method used, with electrostatic spinning using a single needle as an emitter and a rotating cylinder as a collector.

For the fabrication of the tissue scaffold, the solution was electrospun at room temperature using a 4SPIN instrument (Contipro, Dolní Dobrouč, Czech Republic), using a single needle as an emitter and a rotating cylinder as a collector covered with aluminum foil serving as a substrate, which made it easier to remove the thin layer of nanofibrous material. The configuration used is illustrated in Table 1 and Figure 1. The process parameters for each sample were selected according to current knowledge. The samples were reproducible and fabricated in several sets to provide more accurate results. The parameters were primarily derived from the scientific papers of Li Yuchao et al. [16], Pisarenko Tatiana et al. [24], and He Zhongchen et al. [25].

The electrospinning parameters had to be optimized through experimentation. If the spinning tension is too high, inhomogeneous or ragged fibers may be produced. A higher flow rate and viscosity of the solution can lead to the formation of thick fibers or droplet defects, while too low a flow rate and viscosity can cause thin and brittle fibers. The rotational speed of the collector influences the orientation and arrangement of the nanofibers, where higher rotational speeds can lead to the formation of fibers with a more uniform orientation and better mechanical strength. The temperature and humidity can affect the solvent evaporation, fiber drying, and defect formation [24]. After achieving a suitable configuration for the application, two sets of samples were fabricated to examine the effect of the nanofiber alignment on cell growth in the tissue scaffold. The rotation speed of the collector was varied between the two sets. These samples were subsequently investigated using SEM. In addition to determining the optimal chosen electrospinning configuration, it was also confirmed that increasing the collector speed from 300 to 2000 rpm reduced and homogenized the nanofiber diameter, reduced the porosity, and orientated the nanofibers in a given direction, providing better support for the cell microstructure. More information on this matter is available in Section 3.1.

### 2.3. Surface Modification Using Plasma

NANO Plasma Cleaner (Diener electronic, Ebhausen, Germany) was selected for etching. In biomedical applications, Ar and O_2_ are typically used, the main difference being the surface activation mechanism, where the use of Ar plasma mainly results in physical etching, but O_2_ plasma increases the surface tension of the material through the removal of contaminants and the introduction of polar molecules.

Given our findings, a modification of part of the specimens was made according to the works of M. Kitsara [23] and D. M. Correia [26] and their collaborators by using O_2_ plasma, Ar plasma, or O_2_+Ar plasma for 2 min at 200 W or 10 min at 300 W. The resulting samples prepared for analysis and cell seeding are listed in Table 2.

### 2.4. Selection of Bone Cells

The human osteosarcoma cell line Saos-2 (HTB-85, ATCC, Manassas, VA, USA) was isolated from the vernacular bone tissue of an 11-year-old Caucasian girl in 1975. The activity of Saos-2 cells, i.e., the expression of cytokines and growth factors and the structure of synthesized collagen, is highly similar to that of osteoblasts. As a result, the Saos-2 lineage is considered to be the most representative cell model used to study interactions in tissue culture media, providing the advantage of rapid and straightforward cultivation [27]. Therefore, Saos-2 cells were used for the present work according to the above findings.

#### Population of the Scaffold by Cells

The cell cultivation processes and the handling of the cells themselves were based on previous research, general culture protocols [28], and the work of Hernandez-Tapia et al. [29].

The culture medium DMEM (Corning, New York, NY, USA), supplemented with 10% fetal bovine serum (Corning, New York, NY, USA) and a combination of antibiotics in the form of a 5% solution of penicillin and streptomycin (50 IU mL^−1^ and 50 g mL^−1^), was used to maintain the obtained Saos-2 cells. Cells were stored in a culture flask for adherent cells with a filter cap at 37 °C in an incubator with humidified air and 5% CO_2_. A trypsinization process using a 0.25% trypsin–EDTA solution was used to extract the cells. Cells were seeded at a density of 1 × 10^3^ cells/mL onto established sterile samples (UV sterilization boxes) of electrospun PVDF nanofibers prehydrated with a culture medium and placed in an incubator for 24 h. Paraformaldehyde-fixed cells were permeabilized with a 0.5% solution of Triton-X 100 and a 2% solution of bovine serum albumin, allowing the dyes to penetrate the cells. Subsequently, the cells were washed thoroughly in deionized water and stained with DAPI to visualize the nuclei and FITC to visualize the cell membrane. All chemicals used were purchased from Sigma Aldrich (St. Louis, MO, USA).

### 2.5. Methods for Fundamental Analysis

#### 2.5.1. Scanning Electron Microscopy (SEM)

Observations were made on an LYRA3 microscope (Tescan, Brno, Czech Republic), using an SE detector for surface analysis. The accelerating electron voltage was 2 kV, the working distance was 9 mm, and the magnification and field of view are given. An attached X-Max 50 EDS detector (Oxford Instruments, Oxford, UK) was used for chemical analysis with an accelerating electron voltage of 5 kV.

#### 2.5.2. Raman Spectroscopy

The three main crystalline phases (α, β, and γ) of the studied PVDF nanofibers were identified from Raman spectra using the WITec Alpha 300R instrument (WITec, Ulm, Germany). A green laser with a wavelength of 532 nm and a power of 5 mW was used during the spectroscopy. The number of accumulations was 10 with an integration time of 10 s using a 50× magnification objective.

#### 2.5.3. Fourier Transform Infrared Spectroscopy (FTIR)

An FTIR Vertex 70s (Bruker, Billerica, MA, USA) with a 4 mm slit in the transmission mode was used for the measurements. The resulting spectrum represented the difference between the sample spectrum, the mean of 16 scans, and the background spectrum, the mean of 32 scans. The measurement was then followed by post-processing. A baseline correction was made to correct the spectra and invert them to obtain the absorbance instead of the transmittance. The spectrum with the most relevant region (1500 to 400 cm^−1^) was then plotted.

#### 2.5.4. Measuring the Contact Angle of the Liquid

The contact angle measurement was based on Young’s equation. See System E (Advex Instruments, Brno, Czech Republic) was used to perform the measurements, and See 7.0 software was used to evaluate the contact angle from the photographs. Using a dispensing micropipette, ten 3 μL droplets of distilled water were sequentially applied on the surface of the nanofiber mats. At t=4 s, the contact angle was measured from the captured image using three selected points, which, when connected, formed a circle that followed the shape of the droplet. The resulting contact angle value for each sample was the average of 10 measurements.

#### 2.5.5. Confocal Microscopy

The interaction of cells with PVDF nanofibers was visualized using immunofluorescence. Stained cells were imaged using a Zeiss LSM 880 confocal microscope (Carl Zeiss AG, Jena, Germany) with C-Apochromat 40× and Plan-Apochromat 20× objectives and lasers at 488 nm (for FITC excitation [30]) and 405 nm (for DAPI excitation [31]). Zeiss ZEN 3.10 LITE was used for the software processing and analysis of the scanned images.

## 3. Results and Discussion

### 3.1. The Evaluation of the Surface Structure of Nanofibers

**Figure 2:**
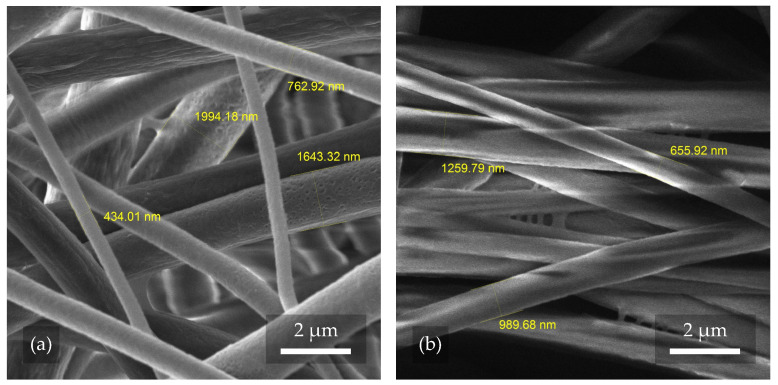
Images of samples 1.1 and 2.1 obtained using SEM. In image (**a**), one can observe chaotically spun nanofibers with a collector rotation speed of 300 rpm, and in image (**b**), one can observe directly spun nanofibers with a collector rotation speed of 2000 rpm.

An examination of the samples—see Figure 2—confirmed that the nanofibers were successfully fabricated without major shape imperfections. The occurrence of droplet defects was found to be minimal due to the correct DMSO/Ac ratio and concentration.

The major difference was, as expected, the arrangement of the individual fibers, which in sample 1.1 (see Figure 2a) were very chaotic, twisted, and misaligned. In contrast, in sample 2.1 in Figure 2b, the fibers can be seen to be aligned and stretched in a relatively similar direction. Another important difference is the thickness of the fibers, which varies considerably in Figure 2a, with the difference between the thickest and thinnest fibers being as much as 1873 nm. In Figure 2b, the thickness of individual fibers is smaller and more consistent, with the largest difference in width being only 604 nm. The higher collector rotation speed led to a more uniform distribution of nanofibers. The lower collector speed caused fibers to clump and become unevenly distributed with more porosity. The same result was also described by Pisarenko et al. [24], when with a comparable instrument configuration, the authors reached the same conclusions. The quantities and fiber diameters were also statistically reported in the mentioned paper. The fiber width and porosity can be controlled in a variety of ways other than the rotating cylinder speeds. The viscosity or humidity of the solution also has a major influence, as mentioned by Medeiros et al. [32,33]. With these choices, the implications that follow should be taken into account, where not only changes in the morphology occur. The following issues are discussed in Section 3.2. Among many other parameters, the collector type has been mentioned as a possible aspect that significantly affects the diameter of the fibers. As reviewed in [33], the standard setting is a plate-shaped collector that serves as a conductive substrate. However, this configuration significantly limits the control over both the diameter of the fibers and their alignment. With the rotating collector used in this experiment, these challenges could be partially resolved.

#### 3.1.1. Morphology of Nanofibers After Plasma Treatment

**Figure 3:**
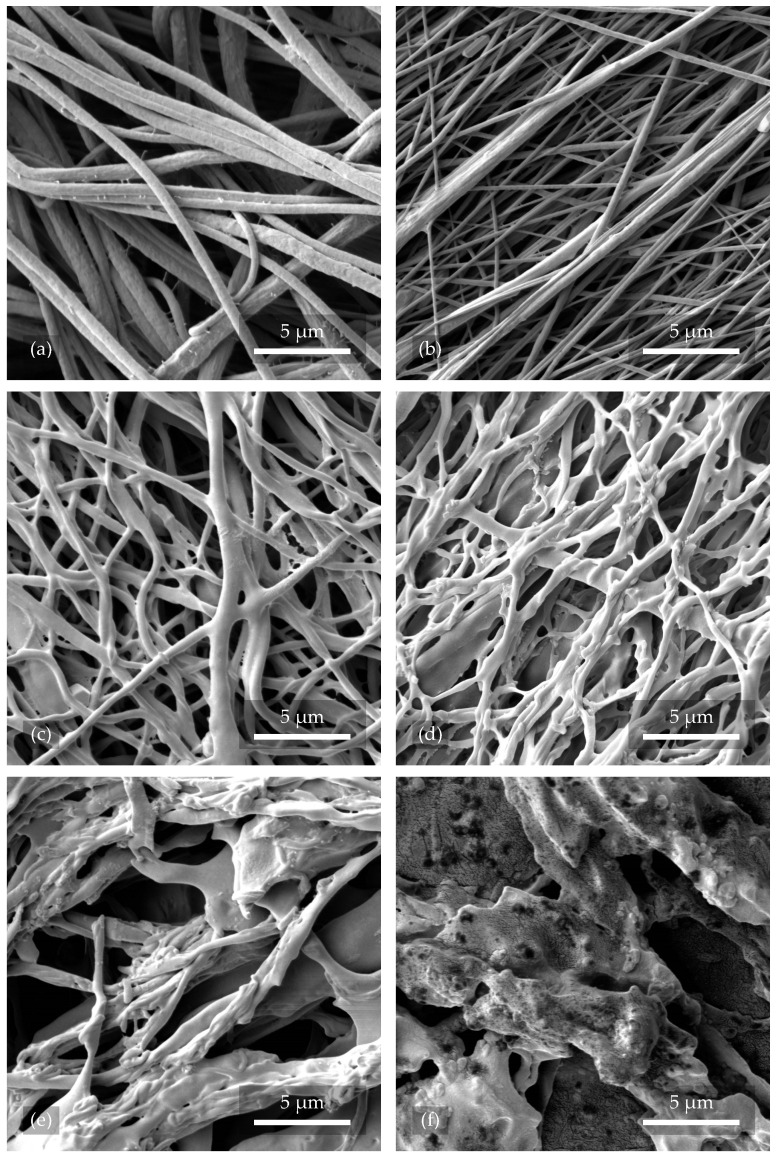
The morphology of nanofibers after plasma treatment at different processing settings, visualized using SEM. Image (**a**) is the reference, while images (**b**–**d**) were treated with plasma at a lower power and shorter etching time and images (**e**,**f**) at a higher etching power.

A reference sample of nanofibers, 2.1, can be observed in Figure 3a, which was not exposed to any kind of plasma. The fibers were intact and without treatment. In Figure 3b, sample 2.3, treated with Ar plasma for 2 min at 200 W, can be seen, where there was no significant change in both the surface and orientation of the nanofibers. However, Figure 3c shows nanofibers from sample 2.4 that were exposed to O_2_+Ar plasma for 2 min with a power of 200 W. Their surface structure was distorted and disrupted, leading to cross-linking and decreasing the average pore size of the whole structure. A similar result can be observed in Figure 3d with nanofibers from sample 2.2. The fibers were treated with pure O_2_ plasma with the same working parameters and experienced the most significant changes in the form of twisting, shape distortion, and coupling. This phenomenon resulted in an increase in the porosity and permeability of the resulting tissue scaffold. As can be seen from Figure 3e,f, the uniformity of the fibers was considerably affected by a longer plasma etching time (10 min) and higher power (300 W).

Compared to the research by Correia et al. [26], where the authors etched fibers with O_2_ plasma at 240 W and for 2 min, our results showed much more surface disruption. In Figure 3c,d, the fibers shown were etched with O_2_+Ar and O_2_ plasma, respectively. The power was 200 W and the time 2 min. These differences are probably due to the different gas flows that were used.

#### 3.1.2. Nanofiber Surface Wettability

**Figure 4:**
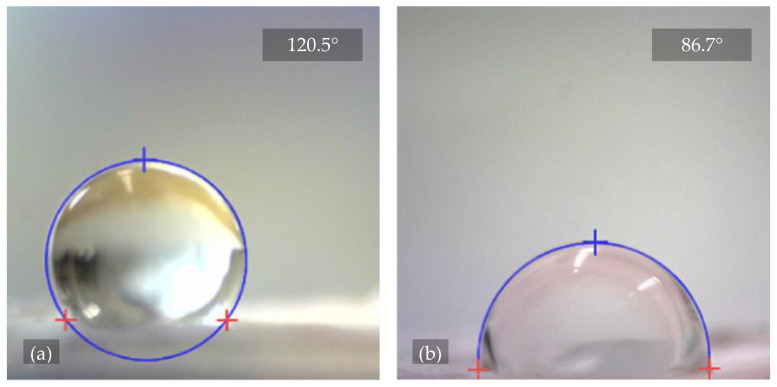
Images from See System E using See 7.0 software to evaluate the contact angle of distilled water on the material surface. The sample images show a three-point measurement, where the hydrophobic and hydrophilic behavior of PVDF nanofibers can be observed in images (**a**,**b**), respectively, in relation to the plasma treatment.

Prior to cell implantation, the PVDF nanofiber samples with unmodified and plasma-modified surfaces from Set 2 were subjected to a liquid surface contact angle measurement. The hydrophobicity interval for the water contact angle on the solid surface was set from 90° to 150° (above 150° being superhydrophobic), and the values in the hydrophilicity interval ranged from 0°–90° [34]. See Figure 4 for sample images of the measurements performed.

The chosen samples were modified with O_2_ and Ar plasma of different treatment lengths and powers to determine their effect on the structure and wettability of the scaffold. Surfaces with a high surface energy compared to the surface tension of the liquid have strong, attractive forces between their molecules, which makes it easier for the liquid to wet the surface. Plasma treatment should, in theory, increase the material’s surface energy, which in practice, should lead to better adhesion properties [35]. The measurements of the contact angle of the liquid on the surface of the material confirmed that unmodified PVDF nanofibers are hydrophobic. Plasma treatment led to a decrease in the contact angle down to the hydrophilic range, with more pronounced effects for O_2_ plasma. Samples treated with plasma under higher operational conditions were found to be unsuitable for application in BTE as they lost their fibrous structure (Figure 3f). The infiltration of cells into such material becomes ineffective as it loses the ability to orient the cells’ growth using fibers.

The surface contact angle of water for the plasma-untreated nanofibers from sample 2.1 had an average value of 124.6°, obtained from 10 measurements at t=4 s. The material did not absorb more liquid until t=600 s. Based on these data, the plasma-unmodified PVDF nanofibers can be classified as hydrophobic.

Further measurements showed that the contact angle of the plasma-treated nanofibers gradually decreased. For sample 2.3, which was exposed to Ar plasma for 2 min at 200 W, the contact angle decreased slightly to 122.2° but was still within the hydrophobic range [34]. However, sample 2.2, treated with O_2_ plasma with the same time and power parameters, showed a significant increase in the surface energy as the contact angle was reduced to an average value of 22.8°, already falling within the hydrophilic interval. The deposited droplets were stable for both samples 2.2 and 2.3 and were no longer absorbed by t=600 s. In general, this difference is probably due to the fact that when inert Ar is used, physical etching and purification (by larger and heavier atoms) primarily occur. The surface chemical structure is not significantly altered, e.g., by introducing O into the bonds instead of F, as in the case of O_2_ plasma (smaller and lighter atoms) [26]. Sample 2.4 can also be described as hydrophilic since it showed a decrease in the contact angle to an average value of 32.5° after exposure to O_2_+Ar plasma for 2 min at 200 W. The deposited droplets were also stable. For sample 2.6, treated with Ar plasma for 10 min at 300 W, the deposited water droplets were immediately absorbed; thus, a zero contact angle value can be assigned to this sample and it can be classified as hydrophilic. The conclusions drawn from the measurements are summarized in Table 3.

Research [23,26] has shown that the surface modification of PVDF nanofibers using plasma is desirable due to improved adhesion properties. Such a modified surface is also reflected in a greater degree of wettability of the material due to, for example, the introduction of oxygen into the bonds and the release of fluorine (defluorination to reduce the hydrophobicity). The wettability results published in this experiment also correlate with the work of Correia et al. [26]. The authors claimed that in the case of O_2_ plasma and a power of 360 W, rapid water absorption had already been observed. In the case of the presented experiment in this paper, similar results were also obtained from the samples used (see Table 2 and Table 3). Therefore, it can be confirmed that even a shorter exposure of the fibers to etching has a large effect on their absorption. Applying O_2_ and Ar plasmas to various polymer substrates has yielded promising results in promoting cell growth. Plasma treatment usually leads to some degree of polymer surface degradation, depending on its type, exposure time, and power. Accompanying the degradation process are the cleavage of molecular chains, the deployment of functional groups, and the formation of free radicals that activate the polymer surface, which is desirable in the context of surface functionalization [23]. It can, however, be possible to achieve hydrophilization without polymer chains rupturing and the disordering of the crystal structure by using a lower plasma power of 120 W, as demonstrated by Kormunda et al. [36]. The authors also reported an increase in the electroactive β-phase content with these parameters.

### 3.2. Crystalline Phase Content

PVDF has a structure in which two H or F atoms are bonded to a C atom and arranged in repeating chains. This structure exhibits charge asymmetry due to the opposite spatial arrangement of H and F atoms with highly different electronegativities. In this case, it is attributed to a non-zero internal dipole moment and is referred to as polar. This arrangement is observed in PVDF as the β-phase. The phase transformation of non-polar α to polar β is achieved by applying mechanical stress to PVDF samples to align the polymer chains and induce a parallel chain conformation (TTT). When placed in a high-voltage electric field, the randomly oriented PVDF molecules become polarized, meaning that their internal dipole moments are arranged in a particular direction. It is the material with a high content of the β-phase that exhibits the strongest residual polarization after removal from the electric field. Electrospinning in the material fabrication process simultaneously induces both mechanical stretching and electric polarization in a single processing step [37].

Although FTIR is a widely used spectroscopic method for characterizing the structure of materials, in the case of PVDF and especially the crystalline phases, many instances in the literature using absorption spectra (sometimes even by the same authors) have come to different conclusions, especially regarding the resolution of the fractions of β and γ of the total electroactive phase around the bands 840 cm^−1^ and 510 cm^−1^. This is usually due to the fact that the individual PVDF samples for a given scientific work were obtained from different solutions with different properties or different fabricating methods and experimental conditions. On the other hand, it has been found that bands of about 763 cm^−1^ and/or 614 cm^−1^, 1275 cm^−1^, and 1234 cm^−1^ can be reliably used to resolve and identify all three major crystalline phases of α, β, and γ [38,39].

FTIR results are now commonly used in addition to the qualitative evaluation of the presence of crystalline phases and also to quantify the content of individual phases in PVDF to estimate its piezoelectric properties. As shown and explained in work from X. Cia et al. [39], the unique bands around 1275 cm^−1^ and 1234 cm^−1^ for the β- and γ-phases could be used to quantify the components of the electroactive phase as part of a universal procedure. The first step is to quantify the α- and total electroactive phase using the bands around 763 cm^−1^ and 840 cm^−1^ (see Equation (1) from X. Cia et al. [39] and A. Zaszczyńska et al. [40]):

(1) $$F_{\mathrm{EA}}=\left(\frac{A_{\mathrm{EA}}}{\frac{K_{840}}{K_{763}}\times A_{\alpha }+A_{\mathrm{EA}}}\right)\times 100\;\%,$$

where $F_{\mathrm{EA}}$ represents the relative content of the components of the electroactive phase (β and γ); $K_{763}$ and $K_{840}$ represent absorption coefficients; and $A_{\alpha }$ and $A_{\mathrm{EA}}$ represent absorbance values for the corresponding phases at 763 cm^−1^ and 840 cm^−1^. The values of the absorption coefficients are $6.1\times 10^{4}$ cm^2^ mol^−1^ for $K_{763}$ and $7.7\times 10^{4}$ cm^2^ mol^−1^ for $K_{840}$.

Subsequently, the peak-to-valley height ratio (P2VHR) of the characteristic peaks around the values 1275 cm^−1^ and 1234 cm^−1^ can be used to express the representation of the individual components of the electroactive phase (see Equations (2) and (3) from X. Cia et al. [39] and A. Zaszczyńska et al. [40]):

(2) $$F\left(\beta \right)=F_{\mathrm{EA}}\times \left(\frac{\Delta H_{\beta }}{\Delta H_{\beta }+\Delta H_{\gamma }}\right)\times 100\;\%,$$

(3) $$F\left(\gamma \right)=F_{\mathrm{EA}}\times \left(\frac{\Delta H_{\gamma }}{\Delta H_{\beta }+\Delta H_{\gamma }}\right)\times 100\;\%,$$

where F(β) and F(γ) represent the percentage of the fractions of β and γ of the total electroactive phase $F_{\mathrm{EA}}$, and $\Delta H_{\beta }$ and $\Delta H_{\gamma }$ are the height differentials (absorbance differentials) between the respective peaks around 1275 cm^−1^ and 1234 cm^−1^ and their closest valleys.

**Figure 5:**
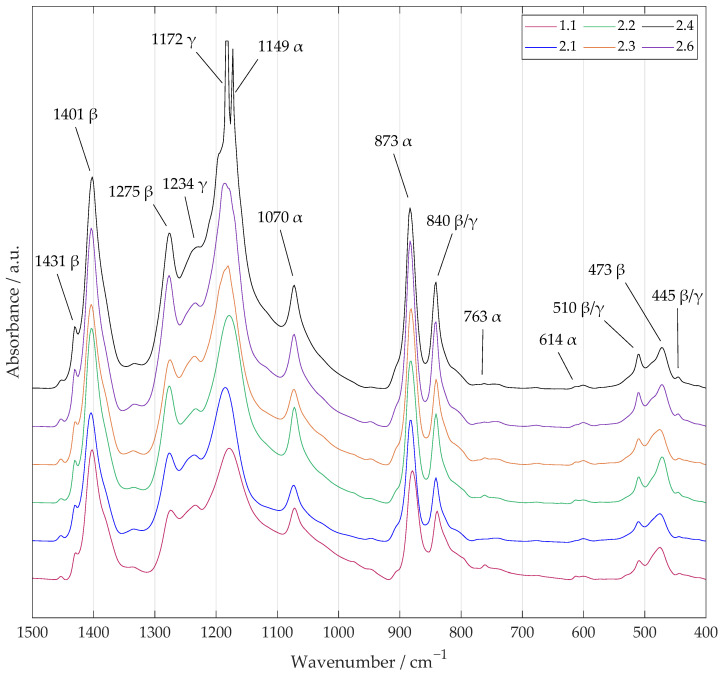
An absorption spectrum obtained using FTIR, cropped to a relevant spectrum of 1500 to 400 cm^−1^. The three basic crystalline phases of PVDF (α, β, and γ) were assigned characteristic and complementary peaks for samples 1.1, 2.1–2.4, and 2.6. The occurrence of the peaks and their ratio with respect to the plasma treatment were studied.

The characteristic peaks of the main crystalline phases in the range of 1700 to 300 cm^−1^ for samples 2.1–2.6 were determined through the comparative study and summary of the FTIR vibrational bands for PVDF from reports published in the current literature and are indicated in the obtained absorption spectra (see Figure 5). The presence of unique bands around 840, 763, 1275, and 1234 cm^−1^ proves the existence of all three basic crystalline phases, α, β, and γ. The classification of the other peaks is only complementary.

In typical samples of semi-crystalline PVDF, the content of crystalline structures is known to be no more than 50 to 60%. However, there is still no specific information on how the amorphous part of PVDF affects the EDS and FTIR spectra. Therefore, the discussion in this work focuses on the critical crystalline phases in terms of possible electroactivity through the piezoelectric effect [39]. Using the above relations (Equations (1)–(3)), the percentage representation of each crystalline phase was calculated and is reported in Table 4.

In all the samples from Set 2, the proportion of the electroactive phase reached at least 90% of the total crystalline phase content, whereas in the sample from Set 1, the value of $F_{\mathrm{EA}}$ only reached 79%. Thus, it can be stated that electrospinning with a higher collector rotation speed has been confirmed as a suitable method for the fast and simple production of PVDF nanofibers with a high electroactive phase content. Furthermore, it was shown that the treatment of the samples with both O_2_ and Ar plasmas at the indicated settings did not significantly decrease their content, although a destructive nature could be expected. However, a change in the ratio of their fractions could be observed to some extent, with a slight decrease in the β-phase to 70% (from the original 77.6% for sample 2.1) at the cost of an increase in the α- and γ-phases for sample 2.3, treated with Ar plasma with a lower power. However, the same phenomenon did not occur for sample 2.6, treated with Ar with a higher power, where, on the contrary, there was an increase in the β-phase to 88.8%. The largest increase in the β-phase occurred in sample 2.4, treated with O_2_+Ar plasma, up to 94.3%, predominantly at the cost of the γ-phase, whose proportion decreased to 0.5%. These results thus support the aforementioned statement by Kormunda et al. [36]. The authors claimed that an increase in the β-phase and crystallinity (which led to better chain ordering too) was also caused during plasma treatment when the sample was exposed to a strong electric field. A natural polarization of the fibers was therefore induced. Conversely, a decrease in the β-phase can occur during C-F bond ruptures and chain scission [36].

Both unmodified samples, 1.1 and 2.1, differing in terms of the rotation speed of the collector during electrospinning, were subjected to Raman spectroscopy, which provided information about the chemical bonds in PVDF and showed the difference in the occurrence of crystalline phases between the samples in the form of characteristic vibrational transitions. These vibrations reflect the bonding arrangement of the atoms in the material, which affects its physical and chemical properties, such as the piezoelectricity. To better understand vibrational transitions, a molecule can be imagined as an elastic structure in which atoms are connected by elastic bonds that allow the atoms to vibrate, rotate, or flap in different ways. As a consequence of the fact that not all symmetric and asymmetric sections are reliably imaged by both FTIR and Raman spectroscopy, the given method is considered complementary for the closest possible description of the crystalline phases of a compound (the detection of additional peaks). Raman spectroscopy was not used for plasma-treated samples, as the scanning of heterogeneous samples is complex, and the instrument’s sensitivity is low.

**Figure 6:**
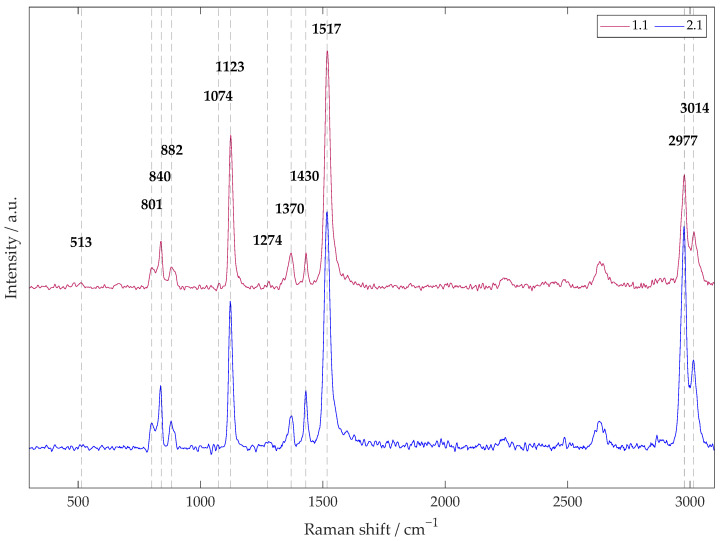
The Raman spectrum in the range of 300 to 3100 cm^−1^ provides information on the vibrational transitions in the analyzed samples, 1.1 (a collector rotation speed of 300 rpm) and 2.1 (a collector rotation speed of 2000 rpm).

The maximum Raman signal intensity (see Figure 6) occurred in the 1517 cm^−1^ region for the vibrational mode of CC coupling and in the CH stretching region (2800 to 3050 cm^−1^), which was dominated by a strong peak at 2977 cm^−1^ belonging to the symmetric mode of the CH_2_ group, all typical for the α-phase. The related peak at 3014 cm^−1^ was associated with the asymmetric stretching of CH_2_ with the β-phase. Other significant peaks were in the regions around 811 cm^−1^ (γ-phase, CH_2_ rocking mode), 840 cm^−1^ (γ-+β-phases, CH_2_ rocking mode and CF_2_ asymmetric stretching), 882 cm^−1^ (α-+β-+γ-phases, CC symmetric and asymmetric stretching and CH_2_ twisting), and 1430 cm^−1^ (γ-+β-phases, CH_2_ twisting + swinging) [41,42]. Sample 2.1, with oriented nanofibers (2000 cm^−1^), showed the same peaks as sample 1.1 but in a larger electroactive-to-non-electroactive ratio and, in addition, showed three more peaks: the first at 513 cm^−1^, assigned to the β-phase; the second at 1074 cm^−1^, characteristic for the β/α-phase; and the third at 1274 cm^−1^, unique to the β-phase [39,42,43]. Thus, the measurement results suggest that the material with directly oriented nanofibers exhibited a higher proportion of the β-phase than the material with chaotically oriented nanofibers. Also interesting is the claim [36] that the higher density and denser chain packing of the β-phase compared to other PVDF crystalline phases results in a reduction in volume, as evidenced in Figure 2 by the decreased fiber diameter and straightened fibers.

### 3.3. The Chemical Characterization of the Tissue Scaffold

The EDS method was performed for the qualitative (elemental distribution) and quantitative analysis (the amount of each element and its ratio) of nanofiber samples 2.1–2.4 and 2.6 (described in Table 2) to confirm the chemical composition of the unmodified reference sample and to compare the effect of plasma treatment on the amount of represented elements expressed in weight percentages and ratios, respectively. A conversion to atomic percentages was not necessary in this case since the difference in the relative atomic weight of the elements present was negligible.

Visualized spectra are more suitable for the identification of elements and the approximate estimation of their amount in measured samples. Elements present in large amounts (>10%) will form high, dominant peaks in the spectrum. In contrast, elements present in minor (1% to 10%) or trace amounts (<1%) will have small or undetectable peaks. A quantitative analysis was therefore performed by software, where the measured signal was compared with the collected data and standards, and the result was a table of mass percentages.

**Figure 7:**
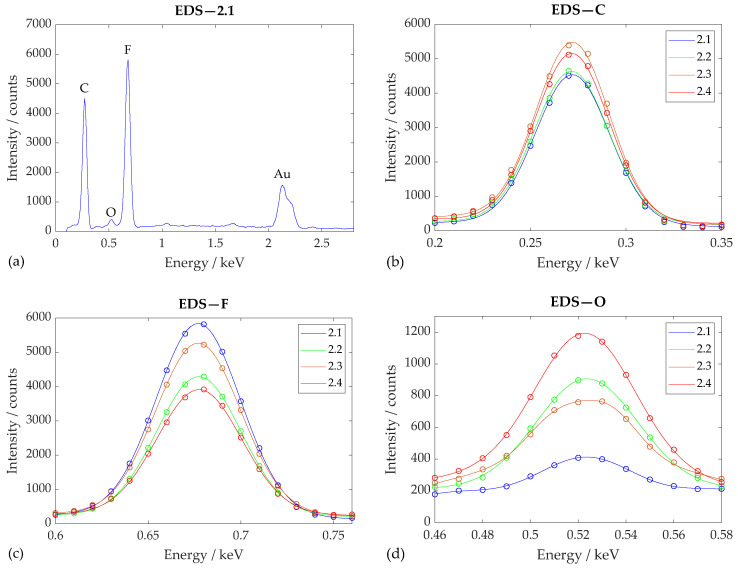
Full EDS spectrum (**a**) and fitted carbon (**b**), fluorine (**c**), and oxygen (**d**). Spectra showing the energies at which X-rays emitted by the samples according to their elemental composition were detected. The recognized peaks are shown according to their respective elements, and the spectra for samples 2.1–2.4 are distinguished by the color; see the legend.

From the energy spectrum of reference sample 2.1 obtained using EDS (see Figure 7), it can be seen that the PVDF nanofibers obtained using the electrospinning method consisted of primarily C (0.277 keV) and F (0.677 keV). PVDF also contains H, which, however, is spectroscopically very difficult to detect since it has only one electron. The presence of relatively small amounts of O (0.523 keV) may have been due to the exposure of PVDF nanofibers to air, particularly during fabrication. Na (1.040 keV) and Cl (2.622 keV) were also detected in small amounts in some other samples of the nanomaterial and can probably be considered as solvent contaminants. A peak for Au (2.120 keV) can also be observed in the spectrum, which is the result of coating the samples with a thin gold film, without which SEM and EDS analysis would be very inaccurate. A small peak can also be observed at an energy of 1.660 keV, but this could not be attributed with any certainty to any element.

Regarding the chemical composition of the nanofibers before and after plasma treatment, it can be noticed (see Table 5) that the mass percentage of carbon indicative of its representation in the sample was relatively stable across measurements around the value of 56%, while oxygen increased from 2.1% in reference sample 2.1 to 8.4% in sample 2.4, etched with O_2_+Ar plasma, and the abundance of fluorine decreased from 45.1°–27.6° in the same sample. This phenomenon was also followed by a decrease in the F/C ratio from 0.856 to 0.599, which allowed us to estimate the defluorination rate at about 30% of the PVDF scaffold. Due to the increase in the O/C ratio from 0.040 to as high as 0.146, the oxygenation rate can be estimated to be about 70% of the scaffold.

For samples 2.3 and 2.6, treated with Ar plasma at different exposure times and with different performances, no significant difference in the percentage of elements could be observed, even if the wettability of the material had changed. This phenomenon can be explained by the fact that Ar plasma treatment leads mainly to physical etching and only causes slight chemical changes.

Changes are also visible in the plotted spectra (Figure 7), where the direction of defluorination and oxygenation in relation to the plasma treatment is also noticeable. The changes in both the F/C and O/C ratios for samples 2.2 and 2.4 indicate the successful nanofiber surface activation of O by plasma in the form of the probable separation of part of the hydrophobic groups C-F and C-H and the subsequent formation of C=O, OH, and COOH hydrophilic functional groups on the PVDF nanofiber surface during the interaction between the plasma and the samples, as shown in the literature [26,44]. According to the authors Jiménez-Robles et al. [45] and Correia et al. [46], the changes they observed in the F/C ratio emerged similarly. The plasma etching caused a decrease in the ratio for the treated PVDF. This may indicate other organic carbon compounds given that the theoretical value of pure PVDF is 1.00, as reported by the mentioned authors.

Analysis using EDS confirmed the chemical composition of PVDF and changes in both the F/C and O/C ratios with respect to the plasma treatment, indicating defluorination and oxygenation, i.e., the successful surface activation of nanofibers treated with just O_2_ plasma. In all samples from Set 2, both untreated and plasma-treated, FTIR showed that the fraction of the electroactive phase was at least 90 % of the total crystalline phases, with the highest representations of the strongly piezoelectric β-phase being for samples 2.2 and 2.4.

The FTIR and Raman results for samples 1.1 and 2.1 confirmed that electrospinning with high collector speeds induces an antisymmetric parallel chain conformation. The mentioned conformation is characteristic for the highly piezoelectric β-phase. This is likely due to the simultaneous mechanical stretching and electrical poles of the electrospun material during the fabrication process. Also, FTIR showed that plasma treatment did not have destructive effects with respect to the content of the electroactive crystalline phase. On the contrary, O_2_ plasma treatment provided the energy necessary to induce the phase transition from the α-phase to the β-phase, introduced polarized functional groups, and stabilized the transformation due to the increase in the surface energy [45].

### 3.4. Investigation of Cell Adhesion

In order to investigate the tissue scaffolds after the seeding, culturing, and fixation of Saos-2 bone cells, SEM was selected. Observations were made with the same configuration as in the previous sections but with a field of view of 350 μm and a magnification of 1600×. The goal was to evaluate the degree of cell colonization of the scaffold. With a field of view of 80 μm and a magnification of 7000×, the goal was to evaluate the shape of individual cells and the character of adhesion.

**Figure 8:**
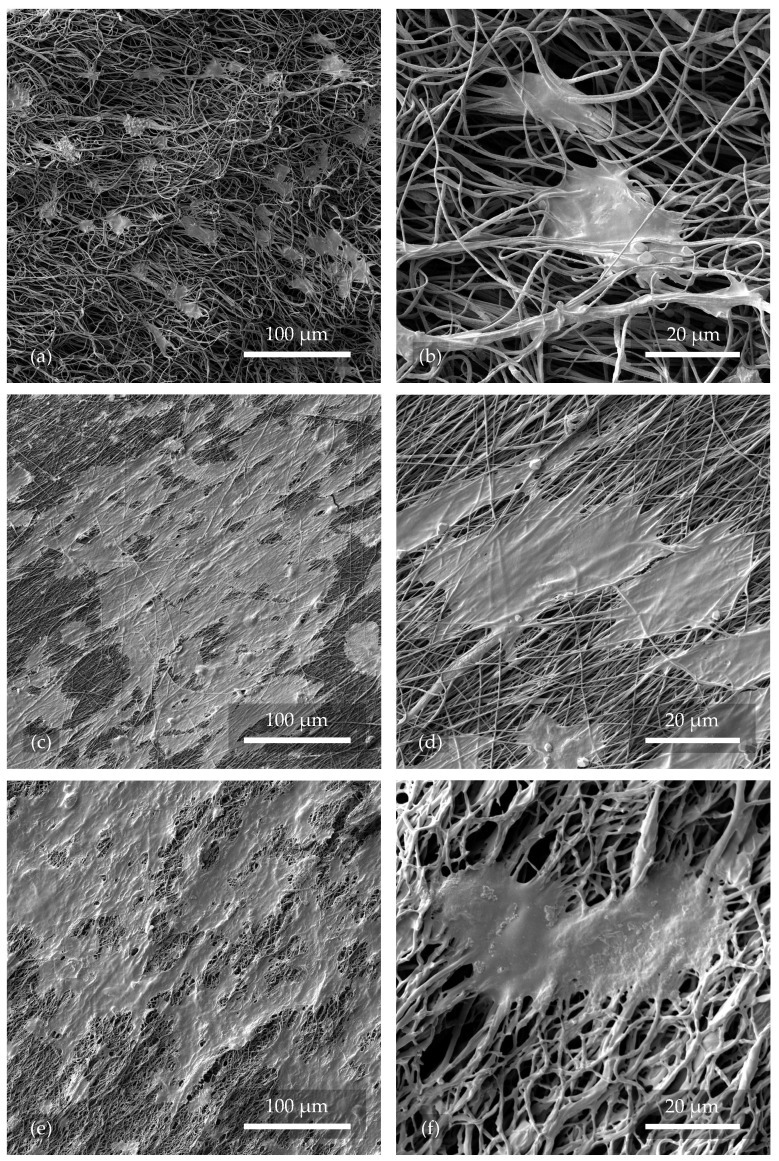
Cell adhesion visualized using SEM. In images (**a**,**b**), there are chaotically oriented nanofibers; in images (**c**,**d**), there are directly oriented nanofibers; and in images (**e**,**f**), there are directly oriented nanofibers treated with O_2_ plasma at lower operational settings (see Table 2).

Saos-2 cells cultivated on nanofibers with a culture medium for 24h were retained to some extent on all samples: 1.1, 2.1–2.4, and 2.6 (see the selected images in Figure 8). Thus, it can be concluded that electrospun PVDF nanofibers left untreated or treated with O_2_ or Ar plasma were biocompatible and non-toxic to the cells used at the given time. Due to the high chemical stability of PVDF, it is not expected to release toxic species into the environment and decrease the cell viability even with prolonged cultivation.

A factor significantly contributing to the success of cell seeding on the nanofibrous material was found to be the higher rotational speed of the cylinder collector in the electrospinning process, which, along with other optimally chosen parameters, provided thin, directly oriented nanofibers with a relatively homogeneous distribution. The fabricated tissue scaffold (see Figure 8c,d) thus provided sufficient support for the cells due to its adequate porosity and, at the same time, provided space for continuous growth and mutual intercommunication. Hence, the cells stretched more on the directly oriented fibers, oriented themselves in the same direction, and formed continuous clusters, which was more advantageous for them in terms of greater reciprocal communication and overall viability. The resulting structure closely mimicked natural tissue. In contrast, in Figure 8a,b, chaotically directed nanofibers can be observed, with only a fraction of successfully implanted cells compared to the directly oriented fibers. Due to the chaotic arrangement of the nanofibers and large pores, many cells probably did not adhere or wholly collapsed due to a lack of support. They were removed during the washing procedure of the samples prior to fixation. The cells were rounder and did not form clusters as well.

In other studies [47], the cells grown on chaotically oriented and plasma-untreated fibers matched those in Figure 8a,b. The culture time of 24 h was also identical. However, the directly oriented and plasma-treated fibers shown in Figure 8c–f achieved a higher coverage. Thus, it is proved that directly oriented fibers have a positive effect on cell growth.

When considering the effect of the plasma on the nanofibers, it was shown that samples 2.3 and 2.6, treated with Ar plasma at both lower and higher operating settings, did not show higher compatibility with Saos-2 cells compared to the reference sample 2.1. Samples 2.2 and 2.4, treated with O_2_ plasma (see Figure 8e,f) and with O_2_+Ar plasma, respectively, showed more promising results due to their demonstrated hydrophilization and the functionalization of their surface by oxygen. The hydrophilic functional groups on the nanofiber surface contributed to improved cell adhesion. The associated higher prevalence of the electroactive β-phase helped through the electromechanical self-stimulation of cell growth and intercommunication [23].

For the following analysis, CLSM was chosen, using two fluorescent dyes, DAPI and FITC. The blue fluorescent DAPI specifically interacts with the A-T rich regions of DNA and is often used to visualize the nuclei of cells in combination with other fluorescent dyes, such as the green fluorescent FITC used here, which passes through the permeabilized cell membrane and binds in the cytoplasm. Because their emission spectra are well separated, the different structures to which the dyes bind [30,31] can be clearly visualized through CLSM.

**Figure 9:**
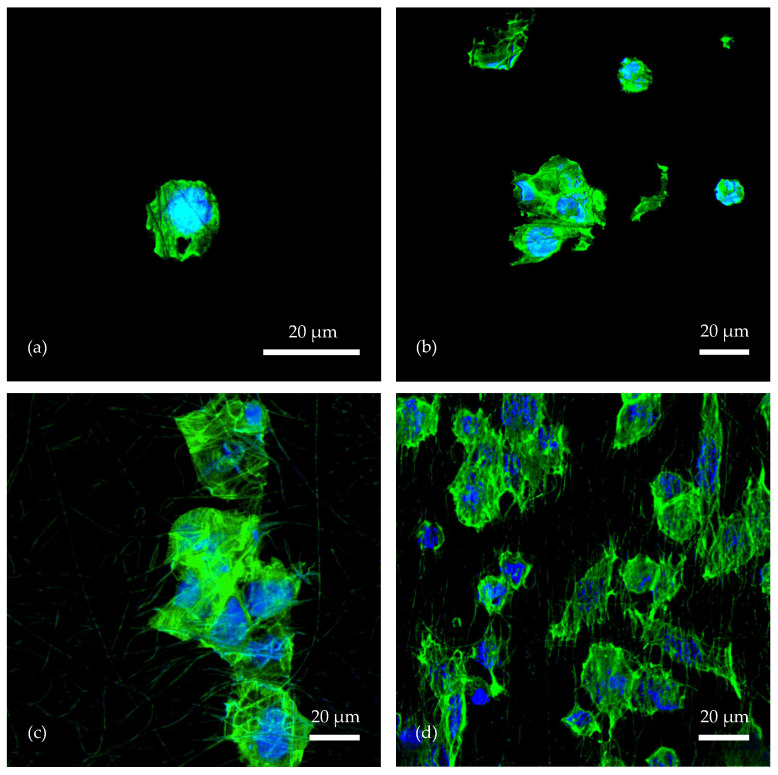
Cells visualized through immunofluorescence and CLSM using fluorescent FITC dyes (binding on the cell surface and in the cytoplasm, emit green light) and DAPI dyes (A-T regions mainly in DNA nuclei, emit blue light). Image (**a**) belongs to sample 2.1, (**b**) to sample 2.3, (**c**) to sample 2.4, and (**d**) to sample 2.2.

The images obtained with CLSM and processed with the compatible ZEN 3.10 LITE software better indicate how the cells deposited on the hydrophilic samples treated with O_2_ plasma penetrated into deeper levels of the tissue scaffold and formed not only surface but also spatially oriented clusters, whereas in the case of hydrophobic samples, the cells remained mainly on the surface. In selected images (see Figure 9), separate blue-stained regions of DNA nuclei can be observed, surrounded by green-stained cytoplasmic cells. The DAPI dye should theoretically also stain the A-T stretches of DNA in the cell’s mitochondria. However, the size of the organelle itself and its DNA stretches is so insignificant compared to the nucleus that they are often not detected at all.

Cell adhesion, or the ability of cells to adhere to surfaces, is a process involving interactions between cell membranes and material surfaces. For tissue engineering and biomedicine applications, the consideration of the many factors that influence this process is essential. Transmembrane receptors, proteins in the ECM, and other adhesion molecules mediate cell adhesion. It depends on the biocompatibility, surface energy, chemical composition, topography, and surface modification of the synthetic nanofibers used to form the tissue scaffold [48].

By studying the fabricated tissue scaffolds using SEM and CLSM, it was shown that the cells were suited to a nanofibrous environment that mimicked the natural fibrous micro- and nano-scale environment of the ECM. The chemical composition of PVDF was found to be non-toxic to and biocompatible with the cells after an overnight culture. Treated nanofiber surfaces with a high surface energy and polarized functional groups improved the wettability and interaction with cell membranes. This led to greater cell spreading over the tissue scaffold surface and into its deeper layers. With respect to this aspect, samples with thin, directly oriented, hydrophilic, and highly piezoelectric nanofibers proved to be the most compatible tissue scaffolds.

## 4. Conclusions

This paper explored potential approaches for designing optimal PVDF fiber scaffolds for bone tissue engineering (BTE). PVDF was selected due to its biocompatibility and suitability for creating functional structures with the desired properties. The primary objective was to fabricate and characterize a biocompatible nanofibrous scaffold for Saos-2 cell implantation.

Through experiments, we optimized the parameters of the fabrication process. Two sets of samples were subsequently fabricated. Fundamental, structural, and functional analyses confirmed the experimental configurations with higher collector speeds of 2000 rpm as being suitable for the construction of a biocompatible tissue scaffold with a high content of the electroactive crystalline phase. The results sumarized several advantages of such an approach. Besides the fact that the nanofibers were more directly oriented, which helped to guide cell growth, they also had a higher β-phase content, which was again an advantage for future piezoelectric stimulation. However, due to the higher flexibility and porosity of nanofibers, the piezoelectric effect cannot be expected to reach the values of solid PVDF layers. Considering the inherent hydrophobicity of PVDF, the nanofibers were treated with O_2_, Ar, and O_2_+Ar plasmas with different exposure times and powers to achieve the most optimal hydrophilic environments for cell adhesion. This method allowed us to efficiently and precisely tune the hydrophobicity of the surface. However, a very aggressive surface treatment process can be expected at high etch rates, especially with large amounts of oxygen, as demonstrated in Figure 8. Osteosarcomatous Saos-2 cells were chosen as the cell material for scaffold colonization, which are considered to be the most representative cellular model for studying osteoblastic-type cell interactions in tissue scaffolds. As a part of the fundamental analysis of the tissue scaffold, SEM was used to investigate the nanofiber quality, surface structure, and cell adhesion. EDS analysis combined with FTIR and Raman spectroscopy indicated a link between the surface structural and functional changes induced by O_2_ plasma treatment. These changes together led to a stable phase transition from the α-phase to the piezoelectric β-phase and surface activation. From the results of water contact angle measurements on the nanofiber surface, the modification of the samples using O_2_ plasma was also shown to be effective in terms of increasing the surface energy and thus hydrophilizing the scaffold. CLSM using the fluorescence contrast from nuclei and cytoplasm staining showed that a significant number of Saos-2 cells attached to individual nanofibers. These cells populated the surface of the scaffold. In the case of hydrophilic electroactive nanofibers, they also infiltrated deeper levels of the scaffold. According to the data collected, samples with thin, directly oriented, hydrophilic, and highly piezoelectric nanofibers fabricated with a collector rotation speed of 2000 rpm and treated with O_2_ plasma for 2 min at 200 W proved to be the most compatible tissue carriers. The electrospun PVDF nanofibers were generally found to be a highly tunable, adaptable, and compatible in terms of their structure. This structure is suitable for application in BTE, allowing for the electromechanical autostimulation of cells due to its piezoelectric properties, which were evident in the crystalline phase contents.

The main limitation of this study was the short-term, 24 h cultivation of the cells on the tissue culture medium before fixation. In future research, it would be useful to more closely study and measure the piezoelectric response of the material as a function of stimulation by an external source of deformation or the adhesion forces of the implanted cells, including a more in-depth analysis of the effects of electromechanical stimulation on the growth, communication, and proliferation of the implanted osteoblastic cells.

## Figures and Tables

**Table 1:** Fabrication parameters of two nanofibrous materials fabricated using the electrospinning method, differing in terms of the setting of the rotation speed of the cylindrical collector. Both materials were sliced into single samples and formed Sets 1 and 2.

Configuration	Set 1	Set 2
Solution	20% PVDF DMSO/Ac	20% PVDF DMSO/Ac
Collector type	rotating collector	rotating collector
Emitter type	1 needle	1 needle
Emitter flow rate	35 μL/min	35 μL/min
High voltage	50 kV	50 kV
Electrode distance	20 cm	20 cm
Collector speed	300 rpm	2000 rpm
Syringe volume	10 mL	10 mL
Ambient temperature	24 °C	24 °C
Ambient humidity	30%	30%

**Table 2:** Order of prepared nanofiber samples named according to the format “set.sample”. The effect of the collector rotation speed during the fabrication of Set 1 and Set 2 was assessed, and the effect of the plasma surface treatment process performed on the samples from Set 2 was investigated. These samples differed in terms of the type of gas used, the power, and the treatment time.

Specimen Number	Modification	Gas Type	Time/min.	Power/W
1.1	No	–	–	–
2.1	No	–	–	–
2.2	Yes	O_2_	2	200
2.3	Yes	Ar	2	200
2.4	Yes	O_2_+Ar	2	200
2.5	Yes	O_2_	10	300
2.6	Yes	Ar	10	300
2.7	Yes	O_2_+Ar	10	300

**Table 3:** A summary of the average values of the surface contact angle for the individual samples from Set 2, with a summary of the wetting characters of the nanofibers, i.e., their hydrophilic or hydrophobic properties.

Sample Number	Contact Angle/°	Wetting Character
2.1	124.6	hydrophobic
2.2	22.8	hydrophilic
2.3	122.2	hydrophobic
2.4	32.5	hydrophilic
2.6	0	hydrophilic

**Table 4:** The quantification of the PVDF crystalline phases using FTIR absorption spectra and the P2VHR calculation method. The results show the percentage representation of each phase (F(α), F(β), and F(γ)) in the respective samples, 1.1, 2.1–2.4, and 2.6.

Content/%	1.1	2.1	2.2	2.3	2.4	2.6
F(α)	21.1	4.1	10.0	9.3	5.2	5.1
F(β)	52.1	77.6	84.3	70.0	94.3	88.8
F(γ)	26.8	18.3	5.7	20.7	0.5	6.1

**Table 5:** A summary of the elemental composition of the prepared nanofiber samples expressed in weight percentages and ratios for chemical characterization according to the plasma surface treatment. Values were derived from the energy and signal intensity.

	Composition/%			Ratio/–	
Sample Number	C	F	O	F/C	O/C
2.1	52.7	45.1	2.1	0.856	0.040
2.2	56.1	34.6	6.8	0.617	0.121
2.3	56.7	37.6	4.7	0.663	0.083
2.4	57.7	27.6	8.4	0.478	0.146
2.6	57.6	34.5	4.7	0.599	0.082

## Data Availability

The original contributions presented in this study are included in the article. The data will be provided on personal request from Nikola Papež. E-mail: papez@vut.cz.

## Abbreviations

The following abbreviations are used in this manuscript:

Ac	Acetone
BTE	Bone tissue engineering
CLMS	Confocal laser scanning microscope
DAPI	4′,6-diamidino-2-fenylindol dihydrochlorid
DMEM	Dulbecco’s modified eagle’s medium
DMSO	Dimethyl sulfoxide
DNA	Deoxyribonucleic acid
ECM	Extracellular matrix
EDS	Energy-dispersive X-ray spectroscopy
FITC	Fluorescein-5-isothiocyanate
FTIR	Fourier transform infrared spectroscopy
PVDF	Polyvinylidene fluoride
P2VHR	Peak-to-valley height ratio
SE	Secondary electron
SEM	Scanning electron microscopy
UV	Ultraviolet
